# Saltation of Non-Spherical Sand Particles

**DOI:** 10.1371/journal.pone.0105208

**Published:** 2014-08-29

**Authors:** Zhengshi Wang, Shan Ren, Ning Huang

**Affiliations:** 1 Key Laboratory of Mechanics on Disaster and Environment in Western China (Lanzhou University), The Ministry of Education of China, Lanzhou, China; 2 Department of Mechanics, School of Civil Engineering and Mechanics, Lanzhou University, Lanzhou, China; Centro de Investigacion Cientifica y Educacion Superior de Ensenada, Mexico

## Abstract

Saltation is an important geological process and the primary source of atmospheric mineral dust aerosols. Unfortunately, no studies to date have been able to precisely reproduce the saltation process because of the simplified theoretical models used. For example, sand particles in most of the existing wind sand movement models are considered to be spherical, the effects of the sand shape on the structure of the wind sand flow are rarely studied, and the effect of mid-air collision is usually neglected. In fact, sand grains are rarely round in natural environments. In this paper, we first analyzed the drag coefficients, drag forces, and starting friction wind speeds of sand grains with different shapes in the saltation process, then established a sand saltation model that considers the coupling effect between wind and the sand grains, the effect of the mid-air collision of sand grains, and the effect of the sand grain shape. Based on this model, the saltation process and sand transport rate of non-spherical sand particles were simulated. The results show that the sand shape has a significant impact on the saltation process; for the same wind speed, the sand transport rates varied for different shapes of sand grains by as much as several-fold. Therefore, sand shape is one of the important factors affecting wind-sand movement.

## Introduction

Saltation plays a key role in various geological processes, including wind erosion, sediment transport, and the formation of sand dunes [1]. In-depth studies of sand saltation, which is the main manifestation of sand movement near the bed surface [2],[3], are considered to be essential to comprehensively understanding wind erosion and desertification processes and, furthermore, to preventing sand disasters[4]. Many scholars have carried out studies to simulate the saltation process [5]–[14]. However, significant discrepancies still exist between the simulated results and measurements [1]. Current models of saltation make many simplifications because of the complexity of sand saltation. For example, most models assume that sand particles are spherical, and the effect of mid-air collision is ignored.

In fact, spherical and sub-spherical sand grains generally comprise no more than 30% of the total sand [15]. Many scholars have studied sand shape in the desert [16]–[20]. For example, Sagga [16] and Juan [17] studied the roundness of sand grains from dunes in Saudi Arabia and Mexico, respectively. Sagga found that the sands of the inter-dunes are more rounded than are those of the adjacent dunes and that sand particles tend to be less rounded with increasing dune height. Juan proposed that aeolian and marine processes might generate different grain sizes with different compositions. Cheng et al. analyzed aeolian sandy physical characteristics and measured the roundness, average particle size and size distribution of desert sand north of Beijing. They indicated that these parameters have an important influence on the interactional force of aeolian sand [18].

Researchers have investigated the mechanical properties of non-spherical particles for over half century. Barton studied the slip correction factors for non-spherical bodies in continuum flow in 1973 [21]. Then, Gavze and Shapiro investigated particles in a shear flow near a solid wall considering the impact of non-spherical grains [22]. In recent years, more studies have been conducted in this field. For example, Yin et al. developed a model of motion of cylindrical particles in a non-uniform flow [23]. Xu et al. discussed drag and lift forces of rotational non-spherical particles [24]. Zastawny et al. considered the drag and lift force and torque coefficients for non-spherical grains in flows [25].

Based on the above studies of the motion of particles in a shear flow, our study first proposes a relationship between sand shape and the threshold friction velocity of wind. Then, a sand saltation model, which takes into consideration the coupling effect between wind and sand grains, the effect of the mid-air collision of sand grains, and the effect of the shape of sand grains, is established, and the saltation process of non-spherical particles is simulated. Finally, the effects of different shapes of non-spherical sand grains on wind-sand movement are analyzed in detail in this paper.

## Methods

### 1. Analysis of sand shapes

Blott and Pye [26] applied Beckman-Coulter to analyze natural sand particles, including desert dune and coastal dune sand particles, and found that the average roundness 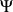 of natural sand is in the range of 0.82–0.86. The average roundness 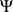 is defined as the ratio of the surface area 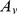 of a sphere to the surface area 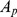 of a sand particle with an equivalent volume, that is
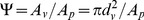
(1)where 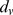 is the equivalent diameter of the sphere with the same volume as that of the sand particle.

Let 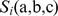 represent non-spherical particles of different shapes, where 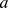, 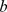 and 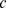 are their size parameters, respectively. In this study, we examined the effects of four types of non-spherical particles, including ellipsoid, cube, cylinder and frustum, on wind-sand movement.

1. Ellipsoid-shaped sand particles: particles with a shape approximate to an ellipsoid are simplified to an ellipsoid, with 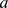, 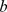 and 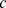 indicating the lengths of the long axis, mid axis, and short axis, respectively. We selected two types of spheroids, 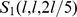 and 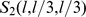, respectively.
2. Cube-shaped sand particles: particles shaped like cubes are reduced to a regular cube 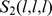.
3. Cylinder-shaped sand particles: particles shaped like cylinders are simplified to a regular cylinder, with 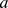 and 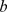 indicating the diameter of the bottom and 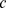 indicating the height. Two cuboids were selected, 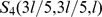 and 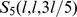; that is, their bottom diameters are *3l/5* and *l*, respectively, while their heights are *l* and *3l/5*, respectively.
4. Frustum-shaped sand particles: irregular frustum-shaped sand particles are reduced to a regular frustum, wherein 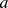, 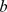 and 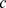 indicate the upper end diameter, the lower end diameter, and the height, respectively. The regular frustum 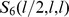 was selected with 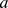, 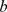 and 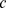 corresponding to 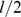, 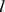, and 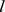, respectively.

The symbol 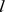 in the above 1) to 4) is a constant. To consider the effects of different shapes, three shape parameters are introduced [27] as follows:

1. The aspect ratio, 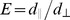, where 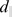 and 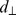 are the maximum diameters or sizes of the horizontal and vertical directions of the non-spherical particles (Table 1);
2. The ratio of the surface areas, 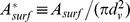; 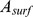 is the surface area of non-spherical grains. By comparison with formula (1), 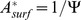 (Table 1);
3. The ratio of the projected areas, 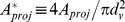; 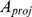 is the projected area of the non-spherical grains (Table 1).

**Table 1:** Parameters of sand particles with different shapes.

	S_1_	S_2_	S_3_	S_4_	S_5_	S_6_
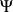	0.853	0.849	0.806	0.847	0.850	0.842
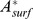	1.1729	1.1779	1.2407	1.1806	1.1765	1.1876
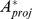	1.8420	0.4808	0.8271	1.0728	0.5429	1.0931
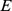	2/5	3	1	3/5	5/3	1

where 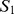 and 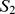 are two different ellipsoid-shaped sand particles, 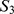 represents cube-shaped sand particles, 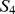 and 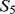 represent two types of cylinder-shaped sand particles, and 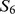 represents frustum-shaped sand particles.

### 2. Drag coefficient of non-spherical grains

The flow field can be divided into three parts according to the Reynolds number [28]: a creeping field of flow (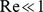), an intermediate field of flow and a Newton field of flow (ca. 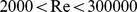).

Under the actions of the three fields of flow, the drag coefficient of spherical particles often is [2],
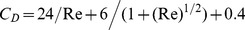
(2)where the Reynolds number 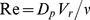.

To consider the impact of particle shape, the modified coefficient of shape is defined as [27],
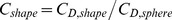
(3)where 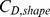 is the drag coefficient of a non-spherical sand particle, and 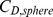 is the drag coefficient of a spherical sand particle.

Under different fields of flow, the modified coefficient of particle shape will be different. The following is an analysis of the shape-modified coefficients of non-spherical particles subject to three types of fields of flow:

The particles in the creeping flow field [27], *Re*<<1:
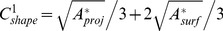
(4)


The particles in the Newton flow field [27], 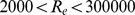:
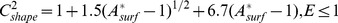
(5)

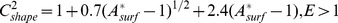
(6)


The particles in the intermediate flow field [27]: 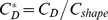, 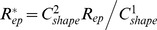, where 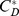 and 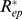 are the substitute of the intermediate flow field. Some scholars have obtained the following empirical formula [27]:

For sand particles with a circular cross-section:
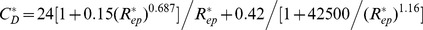
(7)


For sand particles with a non-circular cross-section:
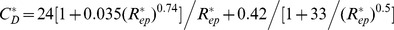
(8)


### 3. Starting friction speed of non-spherical particles

Before starting to move, sand particles on the bed surface are mainly subject to the gravity 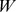, the drag force 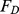, the force of supporting the bed 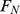, and the cohesive force 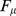 among the sand grains, among which the gravity and the drag force are given as follows [11]:
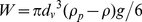
(9)

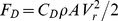
(10)where 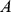 is the maximum area of the particle cross-section. *A* will adopt different values for different sand shapes; 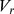 is the relative speed between the sand grains and the gas flow; 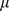 is the aerodynamic coefficient of viscosity; 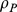 is the density of the sand particles; 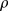 is the density of air; and 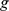 is the acceleration due to gravity.

Here, we focus on dry sand particles. Thus, for large particles, the cohesive force among the particles is very small and can be ignored [2]; that is, 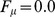.

**Figure 1:**
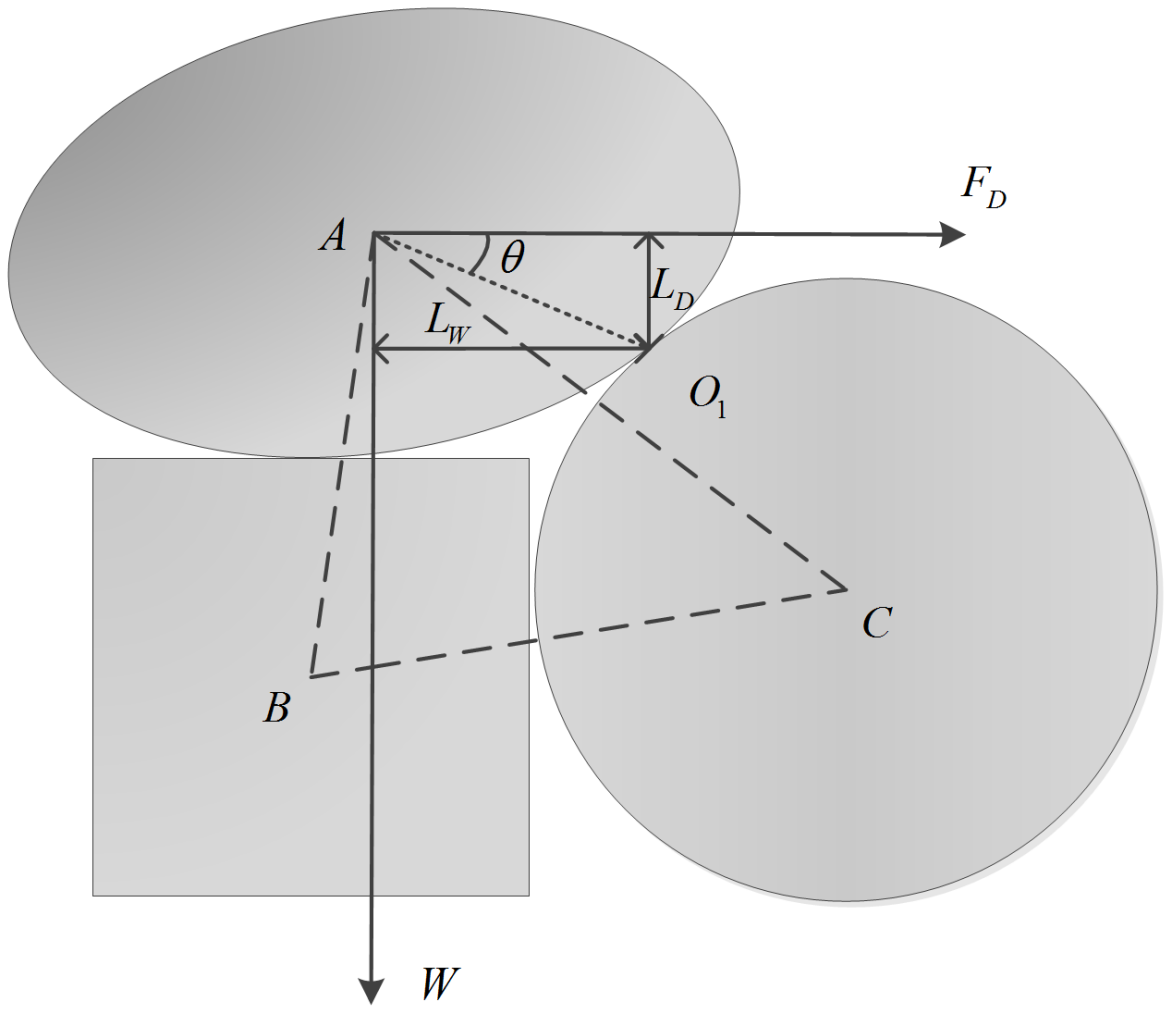
Schematic of analysis of the forces acting on a sand particle. Particles B and C are the supporting sand particles on the bed surface.

Fig. 1 shows a schematic of the force analysis among three non-spherical particles on the bed surface, in which particles B and C are the supporting sand particles of the bed surface. Relative to Point 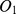, the sand particle A is subject to the moment of force. According to the moment balance principle, as the sand particles satisfy formula (11), they will leave the bed surface:
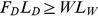
(11)where 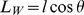 and 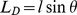.

From inequality (10), the critical condition for the sand particles to start saltation is:
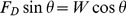
(12)


Substituting formulas (9) and (10) into (12), we obtain the starting speed of flow as follows:
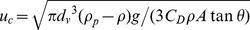
(13)


For a given sand particle shape, 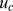 is only related to 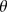, which is a random variable, depending on the acting point of the supporting particle against the saltation particle.

### 4. Wind field equation

Before sand particles start to move near the ground's surface, the motion of air flow can be described by the Navier-Stokes equation:
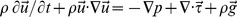
(14)where 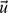 is the velocity of wind, 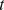 is time, 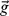 is the gravitational acceleration, 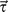 is the Reynolds' tangential stress, and 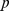 is the pressure.

When sand particles leave the bed and move into the saltation layer, the blocking effect of the particles against the wind field will change their distribution, and this blocking effect can be equivalent to adding a correction term, the volume force, to the right of the Navier-Stokes equations:
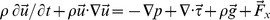
(15)


Letting the direction of air flow movement be in the x-axis and the direction perpendicular to the bottom be the y-axis, 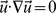, 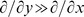, 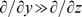. Then, we obtain the motion differential equation of the wind field component in the x direction of in the saltation layer of the wind sand flow.
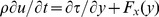
(16)


If a two dimensional steady state is fully developed in the flow field, then the left term in Eq. (16) is equal to zero, that is, 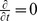; thus, Eq. (16) becomes
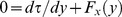
(17)


The blocking effect of saltating particles against the wind field changes with height, and thus the stress acting in the field of the wind also changes with height. Integrating Eq. (17) with respect to height results in Eq. (18):
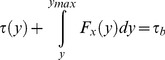
(18)where 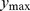 is the max height to which the particle can move upward, 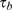 is the shear stress of the air flow from the blocking effect from the sand particle above 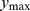, 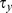 is the air flow shear stress at height 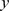, and 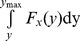 is the particle-loaded tangential stress [7], [11].

Based on the Prandtl mixing length, the Reynolds tangential stress becomes
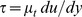
(19)


In the saltation layer of the wind-sand flow, the use of the equivalent friction speed can obtain [7], [11]:
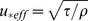
(20)

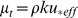
(21)where 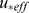 is the equivalent friction speed in the saltation layer and varies with y, and 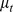 is the viscosity coefficient of the turbulent flow. Bringing Eqs. (19)–(21) into Eq. (18), we obtain the two-dimensional steady state flow field equation fully developed for wind sand flow [13]:
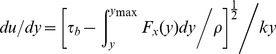
(22)


In accordance with the logarithmic distribution profile that the wind field obeys
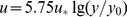
(23)where 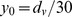 is the roughness of the bed surface.

The critical starting height is 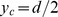. Bringing 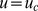 and 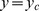 into formula (23), we can obtain the critical starting friction speed 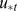 of non-spherical sand particles.

### 5. Saltation equation

The analysis of sand grains subject to forces indicates that in the process of their movement, they experience only the gravity W and the drag force 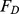, as shown in Formulas (9) and (10), respectively. The basic equations of sand saltation movement are:
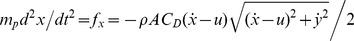
(24a)

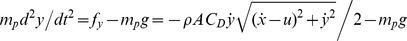
(24b)in which 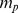 is the mass of the sand particles, g is the gravitational acceleration, 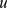 is the wind speed in the x-direction, and 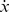 and 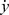 are the velocity components of the particles in the x-direction and y-direction, respectively.

The starting initial velocity distribution of saltating sand particles was obtained by Dong et al. in their experiments [29]:
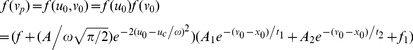
(25)where 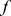, 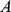, 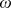, 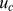, 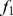, 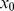, 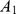, 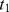, 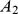, and 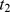 are recession parameters.

### 6. Resistance of saltating sand groups to the wind field

The interaction between the wind flow and the saltation layer plays an important role in the saltation process [3]. After considering the sand shape, the resistance from sand particles experienced by the wind per volume can be described [13]:
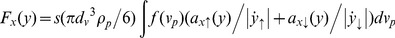
(26)where 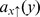 and 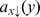 denote the accelerations of the particles moving upwards and downwards at height y, respectively, and 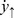 and 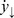 are the average speeds of the particles moving upwards and downwards at height y, respectively.

### 7. Calculations of the sand transport rate (STR) and the STR per width

Considering the mechanism of sand collision, the STRs of irregular particles can be divided into two STRs representing moving particles with collision and those without collision in air. The STR at height *y* considering the aerial collision mechanism of sand particles and the co-initial speed distribution of starting particles is described as [13], [30]:
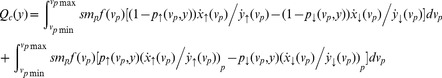
(27)


Integrating Eq. (26) with respect to height, we obtain the STR per width of irregular particles [13], [30]:
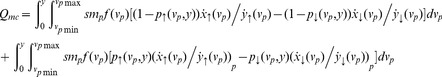
(28)where 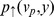 is the probability of collision of rising particles at height 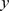 with an initial lifting-off velocity of 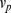, 
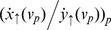
 is the ratio of the horizontal velocity to the vertical velocity after the aerial collision of the rising particles at height 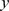 with an initial lifting-off velocity of 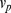 considering the aerial collision mechanism, 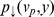 is the probability of collision of falling particles at height
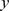
with a co-initial lifting-off velocity of 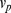, and 
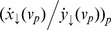
 is the ratio of the horizontal velocity to the vertical velocity after the aerial collision of falling particles at height *y* with a co-initial lifting off velocity 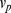.

### 8. Criterion of wind sand flow up to dynamic equilibrium

The critical impact starting velocity of wind is approximately 80% of the starting wind velocity of a critical fluid [2]. From the starting wind speed of a critical fluid 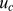 obtained from Eq. (13), the critical impact starting shear stress considering the sand shape is
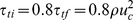
(29)where 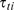 and 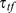 are the critical impact starting shear stress of the wind sand flow and the starting shear stress of a critical fluid [13].

### 9. Constants in the model

For the convenience of comparison, the equivalent diameters of various shapes of sand particles are assumed to be the same. We select two groups of sand particles in this article, d_v_ = 0.25 mm and 0.35 mm; the sand density is ρ_p_ = 2650 kg/m^3^, the air density is ρ = 1.22 kg/m^3^, and the dynamic viscosity coefficient of air is 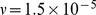; and we assume that the initial wind speed profile obeys a logarithmic distribution.

### 10. Computational steps

1. Substitute the parameters of non-spherical sand grains 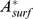, 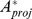 and 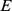 into Eqs. (4)–(8) to calculate the corresponding shape parameters and calculate the resistance coefficients of different shape of particles 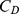, then bring the coefficients into Eq. (10) to obtain the drag forces of different shapes of sand particles 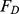.
2. Find the values of parameters 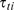 and 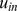 and calculate the initial friction wind speed 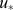, then obtain the initial wind speed distribution and denote them 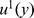 or 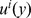.
3. Select the testing value of the sand liftoff rate from the sand bed 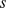, calculate the co-initial speed distribution function of the saltation particles from formula (25), and denote the calculated results 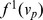 or 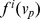.
4. Substitute 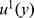 or 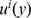 into Eq. (23) to calculate the saltation trajectories of various sand groups and mark the obtained trajectories as 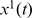 or 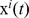, 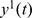 or 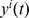, 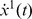 or 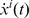 and 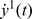 or 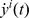. Put 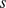, 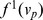 (or 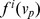), 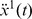 (or 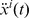), and 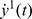 (or 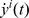) into formula (26) to find the reaction force of the particles at different heights of wind 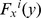.
5. Substitute 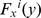 into Eq. (22) to find the wind speed profile modified by saltation particles 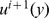, then find the shear stress of the wind acting on the bed surface 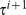.
6. If 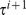 is greater than (smaller than) 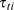, choose the greater (smaller) 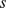 and repeat Steps 3–6 until 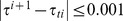.
7. Substitute the values of 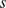, 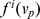, 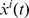 and 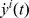 calculated according to Step 6 and satisfying 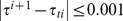 into formulas (27) and (28) to find the STR and the STR per width considering sand shape changes with height.

## Results and Discussion

**Figure 2:**
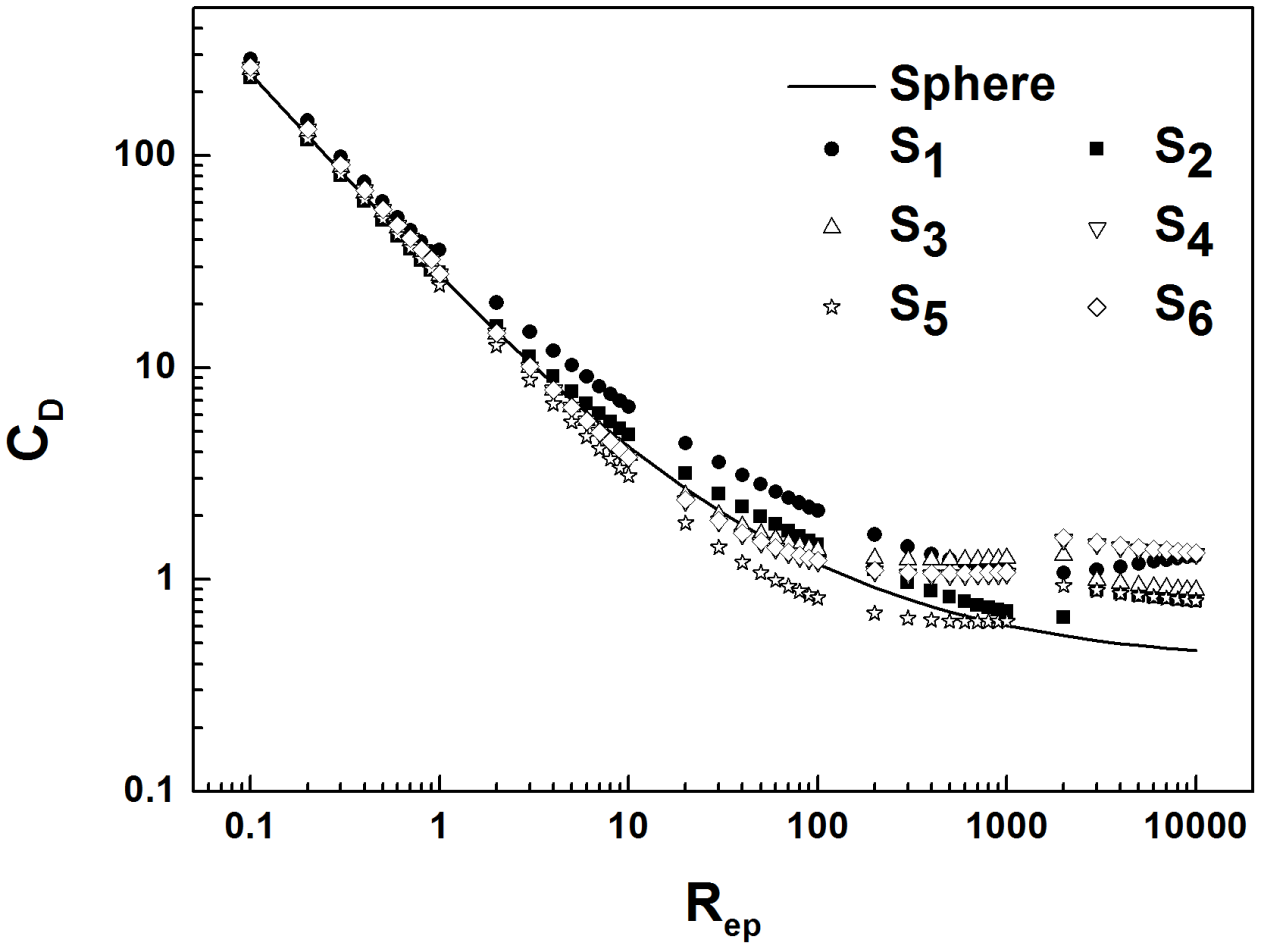
The drag coefficient of sand particles as a function of the Reynolds number. The relationship of the drag coefficient of sand grains of different shape to the Reynolds number is shown. Here, the black solid line indicates spherical sand grains, 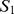 and 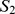 are two different ellipsoid-shaped sand particles, 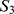 represents cube-shaped sand particles, 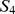 and 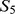 represent two types of cylinder-shaped sand particles, and 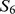 represents frustum-shaped sand particles.

The relationships of the drag coefficients of six non-spherical sand granules to the Reynolds number are shown in Fig. 2. From the figure, it is obvious that the drag coefficients of non-spherical sand particles are greater than those of spherical granules and that the greater the Reynolds number 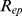, the more obvious the difference between the non-spherical and spherical granules.

**Figure 3:**
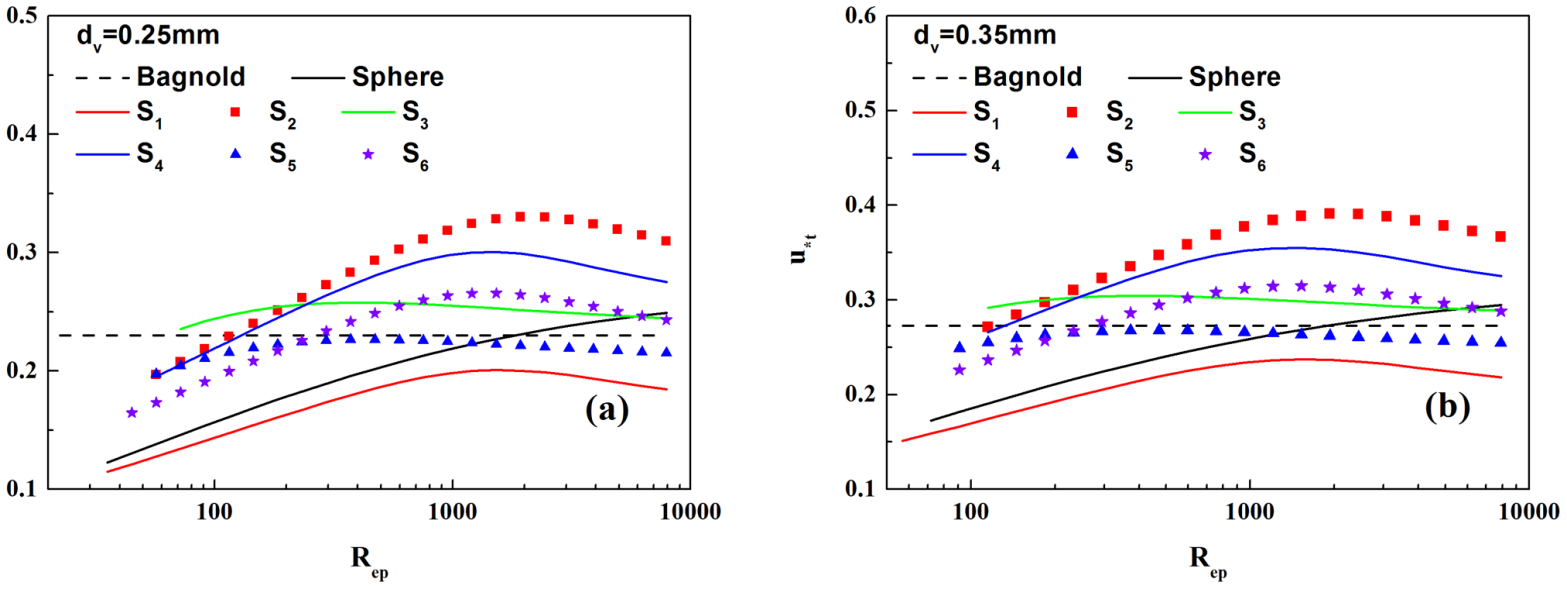
The critical starting friction wind velocity for sand grains of different shape. (a). Comparison of the critical starting friction wind velocity of non-spherical particles with that of spherical particles (equivalent diameter 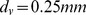). (b). Comparison of the critical starting friction wind velocity of non-spherical particles with that of spherical particles (equivalent diameter 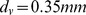). The black solid line indicates the spherical sand grains, 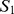 and 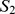 represent two different ellipsoid-shaped sand particles, 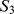 represents cube-shaped sand particles, 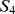 and 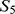 are two types of cylinder-shaped sand particles, and 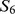 represents frustum-shaped sand particles.

Fig. 3 shows the relationship between the critical starting friction wind speed of non-spherical sand flow and the Reynolds number. It can been seen from this figure, the critical starting friction wind speed given by the Bagnold is a fixed value when the particle size is invariant, while the critical starting friction wind speed in this paper changes with the particle Reynolds number. And the critical starting friction wind speeds for different shapes of sand particles are different for the same Reynolds number. For example, when the Reynolds number 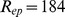, the critical starting wind speed of spherical sand particles is 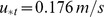 (for D = 0.35 mm) (Fig. 3-b), while the critical starting wind speed of non-spherical particles S_2_ is 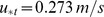 (for D = 0.35 mm) (Fig. 3-b), representing an increase of 35.53% compared to the spherical case. Thus the effect of the sand shape on the critical starting friction wind speed is evident. This is mainly due to the drag coefficient changes with the particle Reynolds number and it is related to the shapes of the particles.

**Figure 4:**
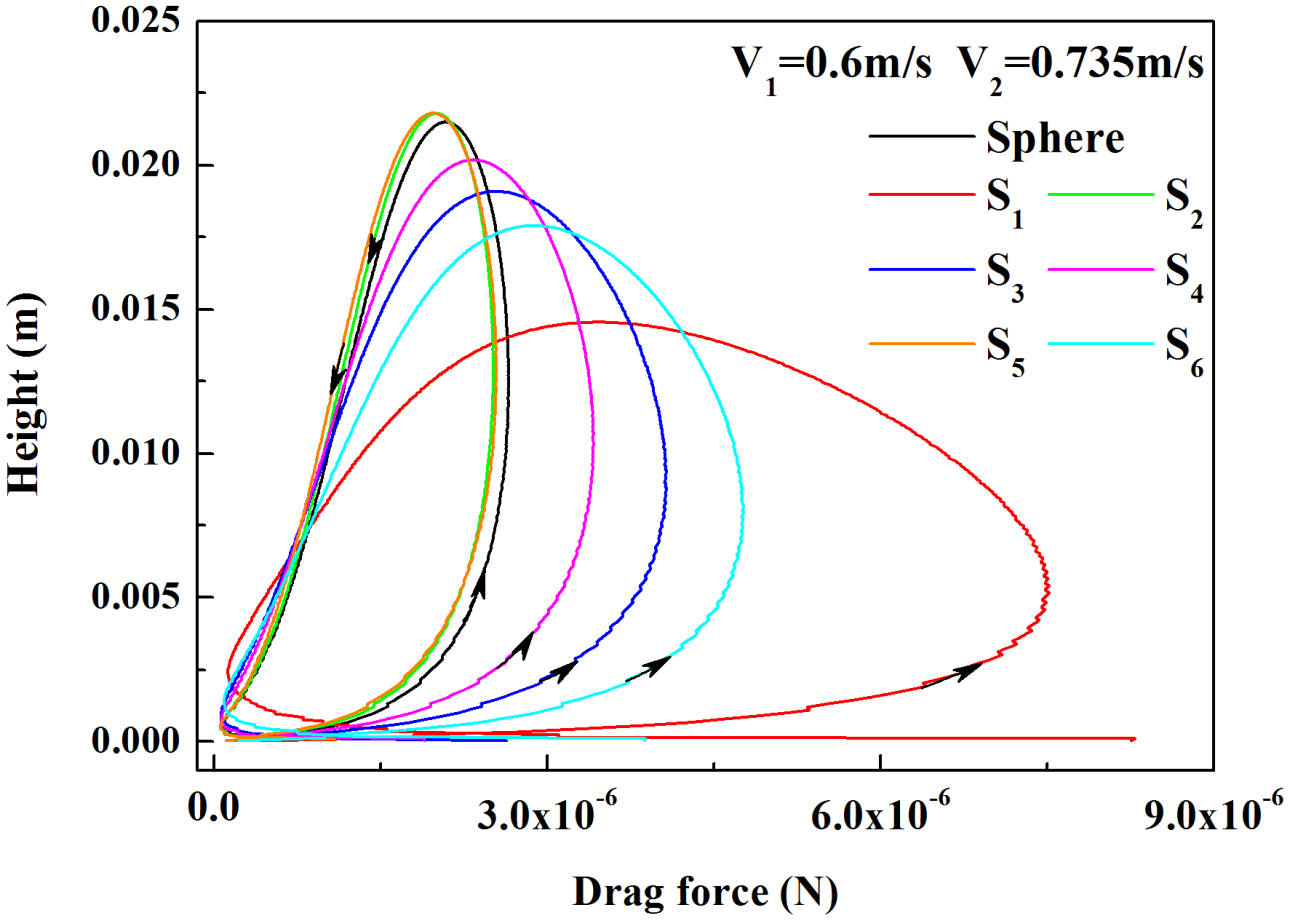
The drag force of different shaped particles with height. All the sand particles of different shapes take off with the same horizontal initial velocity of 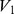 and vertical initial velocity of 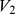. The black solid line indicates spherical sand grains, 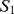 and 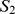 are two different ellipsoid-shaped sand particles, 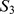 represents cube-shaped sand particles, 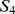 and 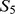 are two types of cylinder-shaped sand particles, and 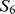 represents frustum-shaped sand particles.

Fig. 4 shows that when the friction velocity is 0.49 m/s and the equivalent diameter is 0.35 mm, the distributions of the drag force are affected by the shapes of the sand particles taking off at an initial speed (such as at a horizontal initial velocity of 0.6 m/s and a vertical initial velocity of 0.735 m/s) along the elevation. It can be observed that during the saltation process, the changes in the drag forces of different shapes of sand particles are consistent, first increasing and then decreasing with increasing saltation height during their ascent, while during their descent, the drag forces first decrease and then increase with decreasing saltation height. Thus, the drag force for different shapes of sand particles at the same height can vary greatly. For example, the maximum drag force of spherical sand particles is 2.65×10^−6^ N, while that of non-spherical sand particles 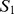 is 7.5×10^−6^ N; the latter is 2.72 times larger than the former.

It can also be observed from Fig. 4 that the drag forces applied to the non-spherical particles whose aspect ratios E are greater than 1.0 are smaller than those applied to the spherical particles, while the drag forces applied to the non-spherical particles whose aspect ratios E are smaller than or equal to 1.0 are larger than those applied to the spherical particles. In addition, the smaller the aspect ratio, the greater the drag force applied to the sand grains and the smaller the maximum height the grains can reach after taking off. Because the drag force suffered by sand grains during their ascent has a vertical downward component, the maximum height they can reach is less than 0.0276 m (the maximum height the freely lifting motion at a vertical initial velocity of 0.735 m/s can achieve is 0.0276 m). The greater the drag force, the greater the vertical downward component and the smaller the maximum height the sand particles can achieve. For example, the drag forces acting upon non-spherical sand particles 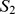 and 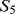 are smaller than those acting on spherical sand particles; the maximum height the spherical sand particles can reach is 2.15 cm, while the non-spherical particles 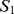 can only reach a height of 1.45 cm. The shape of the sand grains therefore has significant impact on the drag force and the maximum height these particles can achieve. Thus, it also has a great impact on the structure of wind-blown sand.

**Figure 5:**
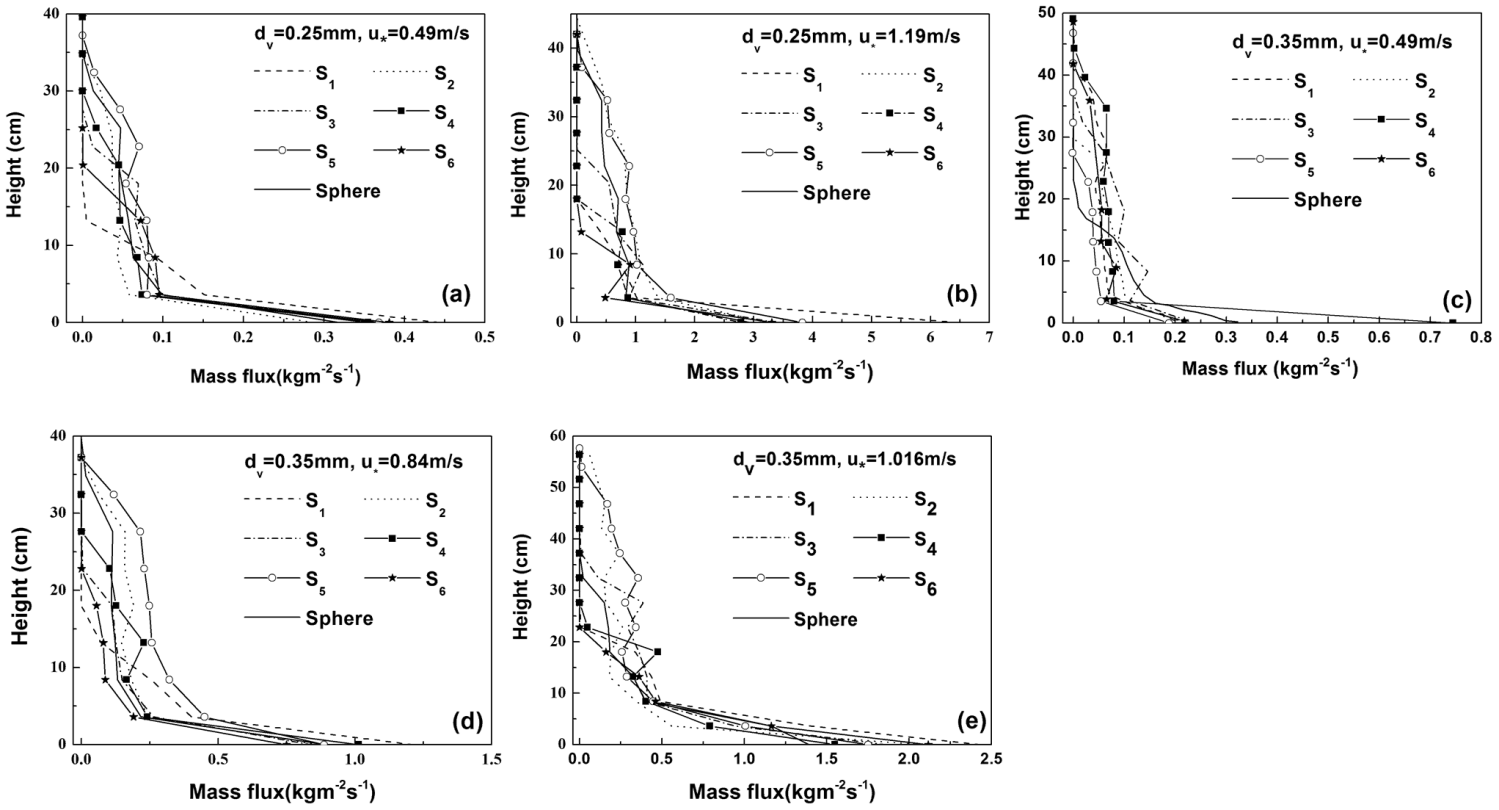
The sand transport rates of sand particles with different shapes as a function of height. (a) and (b) show the sand transport rates of sand particles with the same equivalent diameter of 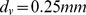 as a function of height but at different friction wind velocities. (c), (d) and (e) represent the sand transport rates of sand particles with the same equivalent diameter of 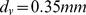 as a function of height but at different friction wind velocities. Here, the black solid line indicates the spherical sand grains, 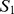 and 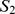 are two different ellipsoid-shaped sand particles, 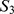 represents cube-shaped sand particles, 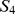 and 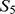 are two types of cylinder-shaped sand particles, and 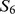 represents frustum-shaped sand particles.

Fig. 5 shows the STR per unit area of different shapes of non-spherical particles with equivalent diameters of 0.25 mm and 0.35 mm as a function of the saltation height. The solid line represents the distribution of the STR per unit area of the spherical sand particles as a function of height. As shown in Fig. 5, the variations in the STR per unit area for different shapes of sand particles with height are equivalent; that is, the STR per unit area decreases with increasing height. However, the STRs per unit area for different shapes of sand particles at the same height vary greatly. In general, the STRs per unit area of the sand particles 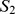 and 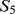 at various elevations are slightly larger than that of spherical sand particles of equivalent diameter. It can also be observed in Fig. 5 that the maximum heights of the wind sand flow formed by different shapes of sand particles at the same wind speed vary greatly, even several-fold. For example, when the equivalent diameter is 0.25 mm and the friction velocity is 0.49 m/s (Fig. 5-a), the sand transport rates per unit area for the non-spherical particles 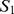, 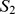, 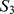, 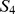, 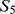 and 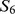 at heights above 13.2 cm, 34.8 cm, 27.6 cm, 30 cm, 37.2 cm and 20.4 cm, respectively, are equal to zero, while that of the spherical sand particles at a height above 34.8 cm is zero. Comparing the non-spherical sand particles 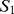 and 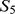, the maximum height of 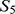 is 2.82 times larger than the maximum height of 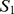.

**Figure 6:**
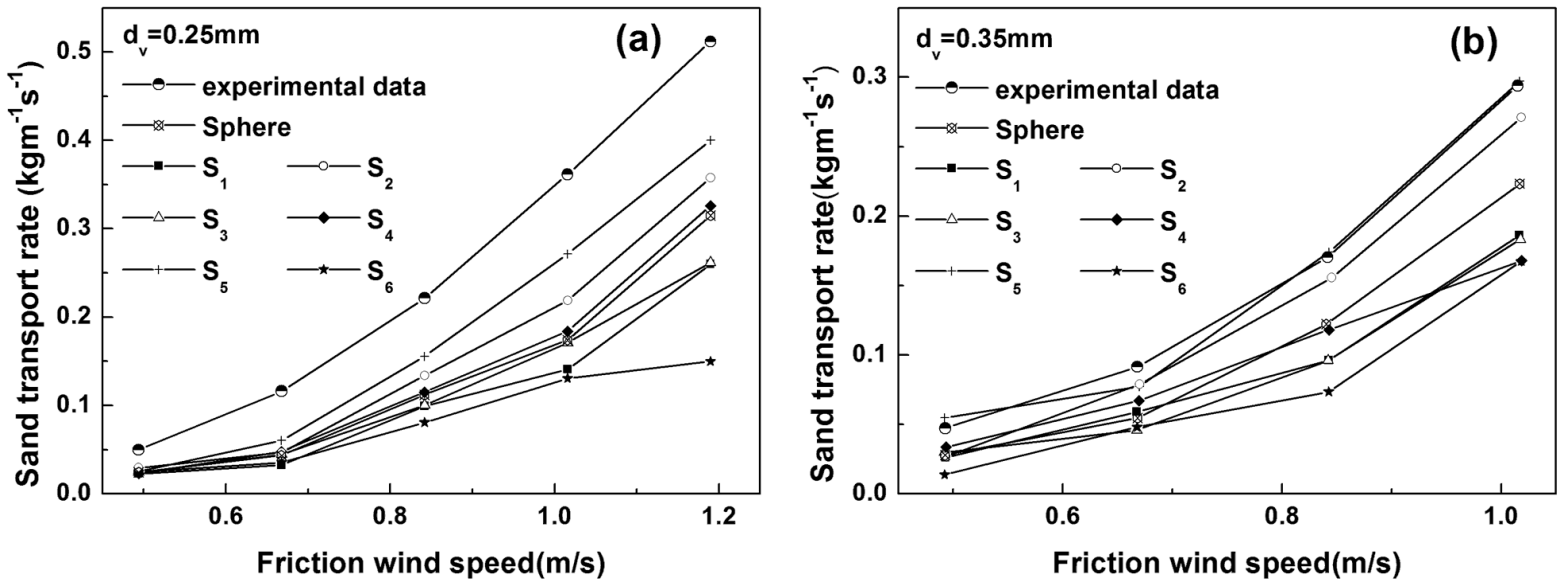
The sand transport rate per width for sand particles of different shape. (a). Comparison of calculated sand transport rates per width for different shapes sand particles with equivalent diameters of 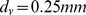 with experimental data. (b). Comparison of calculated sand transport rates per width for sand particles of different shape with equivalent diameters of 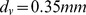 with experimental data. The black solid line indicates spherical sand grains, 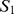 and 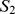 are two different ellipsoid-shaped sand particles, 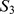 represents cube-shaped sand particles, 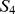 and 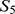 are two types of cylinder-shaped sand particles, and 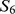 represents frustum-shaped sand particles.

Fig. 6 shows the relationship of the STR per width of non-spherical sand particles to the friction wind velocity, among which Fig. 6-a and Fig. 6-b correspond to equivalent particle diameters of 0.25 mm and 0.35 mm, respectively. It is clear from the figures that the STRs per width for the different shapes of sand particles with friction wind speed exhibit similar variations; that is, they increase with increasing friction speed, in agreement with Dong's experimental results [31]. Carneiro et al. pointed out that the mid-air collisions exert an enormous effect on saltation flux and it depends strongly on restitution coefficient and wind speed [32]. As shown in Fig. 6, the impact of the sand shape on the STR per width is also significant. Under the same conditions, the STR per width for different shapes of sand particles can differ by several-fold. For example, when the equivalent particle size is 0.35 mm and the friction wind speed is 1.016 m/s (Fig. 6-b), the STRs per width of the non-spherical particles 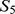 and 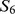 are 0.056 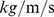 and 0.015 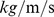, respectively, where the former is 3.73-fold larger than the latter.

Thus, in the discussion of the STR, the sand shape is again an important factor. The main reason is that the air drag forces acting on different shapes of sand particles taking off at the same velocity as well as their maximum heights are different, resulting in large variations in energy when they fall to the bed surface. Thus, the differences in the STR are also significant.

## Conclusions

In this article, we established a relationship between the starting friction wind velocity and the sand shape, simulated the STR and STR per width of wind sand flow reaching the steady state considering the sand shape and the distribution of co-velocities at which the sand particles lift off, and calculated the drag forces and the starting friction wind velocities of different shapes of non-spherical sand particles in wind sand flow. The results show that:

(1) The drag coefficients of non-spherical sand particles are greater than those of spherical granules, The greater the Reynolds number is, the more obvious the difference between the non-spherical and spherical particles.

(2) The change in the drag force is consistent for different shapes and sizes of sand particles; i.e., during their ascent, the drag force first increases and then decreases with increasing saltation height, while during their descent, it first decreases and then increases with decreasing saltation height. The drag force to which the sand particles are subjected is inversely proportional to the maximum height they can reach after their takeoff and closely related to the aspect ratio.

(3) The critical starting friction wind speed of sand particles is not a constant. It changes with the particle Reynolds number. The critical starting friction wind speeds of different shapes of sand granules are different for the same particle Reynolds number.

(4) The STR per unit area as a function of height display similar variations for sand particles of different shapes that is, it decreases with increasing height. However, the STR per unit area for sand particles of different shapes at the same height varies greatly, as does the maximum height that the sand particles can reach at the same wind velocity, which varies even by several-fold.

(5) The STR per width as a function of the friction wind speed is similar for sand particles of different shapes; that is, it increases with increasing friction wind speed. The effect of the sand shape on the STR per width is significant; under the same conditions, the STR per width for sand particles of different shapes can vary by several-fold.
